# Supplementary material for: Impact of Formate Supplementation on Body Weight and Plasma Amino Acids
Source: Nutrients. 2020 Jul 22;12(8):2181. doi: 10.3390/nu12082181 (PMC7469024; doi:10.3390/nu12082181)
Supplement: Supplementary file 1 [file nutrients-12-02181-s001.zip › Supplementary figure 1.pdf]

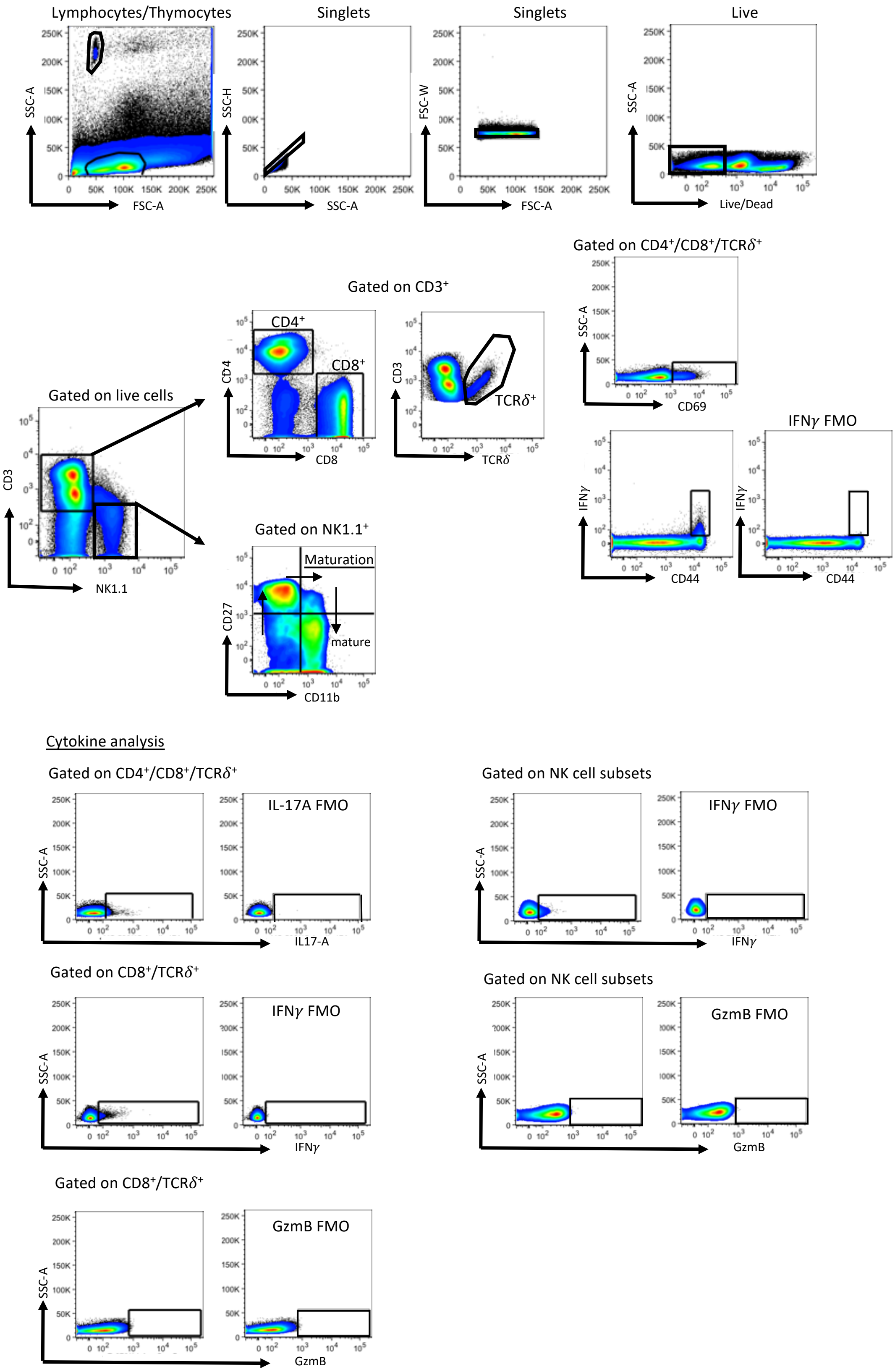

Supplementary figure 1: **Gating strategy for various immune cell populations of blood, thymus and spleen.** Lymphocytes/thymocytes were excluded for doublets twice before gating on live cells, which also excludes $CD19^+$ and $EpCAM^+$ cells. $CD3^+$ lymphocytes and thymocytes as well as NK cells were investigated as depicted. Activation status of T cell subsets (including $CD4^+$, $CD8^+$ and γδ T cells) was investigated based on expression of CD69 as well as CD44 and IFNγ. NK cell maturity was assessed by CD11b and CD27 expression. NK cell subpopulations as well as T cell subsets were analysed for cytokine production as indicated. Fluorescence minus one (FMO) controls were used to facilitate gating.
