# Supplementary material for: Impact of Formate Supplementation on Body Weight and Plasma Amino Acids
Source: Nutrients. 2020 Jul 22;12(8):2181. doi: 10.3390/nu12082181 (PMC7469024; doi:10.3390/nu12082181)
Supplement: Supplementary file 1 [file nutrients-12-02181-s001.zip › Supplementary figure 2.pdf]

Supplementary figure 2

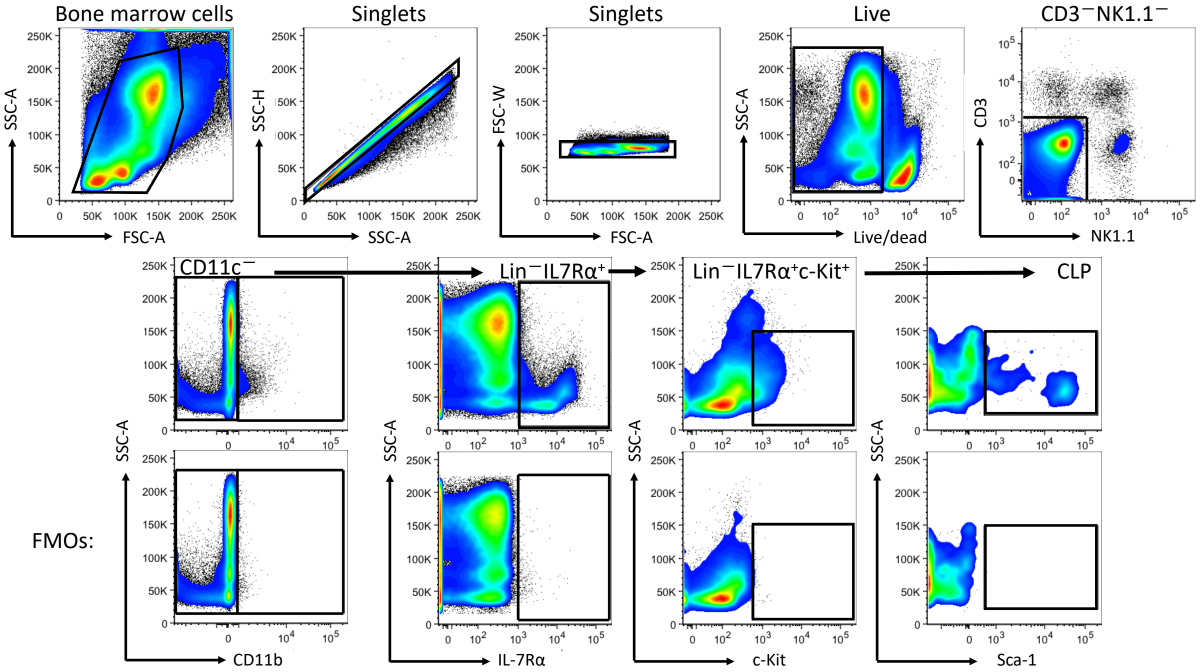

Supplementary figure 2: **Gating strategy for bone marrow analysis.** Bulk bone marrow cells were excluded for doublets twice before gating on live cells, thus further excluding lineage markers such as CD4, CD8, CD11b, CD19 as well as Ter-119. CD11c⁻ cells were considered as lineage-marker negative (Lin⁻) cell population. Common lymphoid progenitor (CLP) cells were characterized as Lin⁻IL7Rα⁺c-Kit⁺Sca-1⁺. Fluorescence minus one (FMO) controls were used to facilitate gating.
